# Melkersson–Rosenthal syndrome: a case report of a rare disease with overlapping features

**DOI:** 10.1186/s13223-018-0316-z

**Published:** 2019-01-05

**Authors:** Mauro Cancian, Stefano Giovannini, Annalisa Angelini, Marny Fedrigo, Raffaele Bendo, Riccardo Senter, Stefano Sivolella

**Affiliations:** 1 Department of Medicine, University of Padua, Padua, Italy; 2 Department of Neuroscience, Division of Dentistry, University of Padua, Via Giustiniani 2, 35128 Padua, Italy; 3 Department of Cardiac-Thoracic-Vascular Sciences and Public Health, University of Padua, Padua, Italy

**Keywords:** Angioedema, Cheilitis granulomatosa, C1-inhibitor, Complement, Melkersson–Rosenthal syndrome, Miescher syndrome, Lip edema

## Abstract

**Background:**

Melkersson–Rosenthal syndrome (MRS) is a rare, neuro-mucocutaneous disease which presents as orofacial swelling, facial palsy and fissured tongue. These symptoms may occur simultaneously or, more frequently, with a oligosymptomatic or monosymptomatic pattern. Swelling, that is the most common initial finding, may mimic hereditary or acquired angioedema, a disorder caused by histamine or bradykinin-mediated plasma-leakage affecting subcutaneous and/or submucosal tissue. The differential diagnosis of MRS includes also chronic inflammatory and infective diseases characterized by granulomatous infiltration, as well as rosacea, contact dermatitis, allergic reactions and Bell’s palsy.

**Case presentation:**

A 71-year old, non-allergic female patient with no familial and personal history of angioedema presented, a few days after a possible herpes simplex or varicella-zoster virus infection, with monolateral facial paraesthesia and lower lip edema. After temporary remission of symptoms on oral steroids and antihistamines, she showed swelling recurrence refractory to valaciclovir therapy and a subsequent course of antihistamines. The clinical picture and a previous history of non-Hodgkin lymphoma prompted us to rule out an acquired form of paraneoplastic, C1-inhibitor (C1-INH) deficiency: C1q and both antigen and functional C1-INH tested normal, whilst we found low plasma levels of C3 and C4 possibly related to the parallel detection of antiphospholipid antibodies. Thus, we hypothesized a non-histaminergic, idiopathic form of angioedema and planned further therapy with tranexamic acid and the leukotriene receptor antagonist montelukast. Treatment failure with both drugs finally suggested a Melkersson–Rosenthal syndrome, which was confirmed by histologic findings of non caseating granulomas on lip biopsy.

**Conclusion:**

Melkersson–Rosenthal syndrome may occur with rather non-specific symptoms and overlap with alternative conditions, including recurrent angioedema. No specific biomarkers for MRS exist and clinical diagnosis is often of exclusion. The finding of complement or immune alterations, as in our patient, may be further confounding and justify the need for skin or mucosal biopsy to establish a correct diagnosis and prescribe targeted therapy.

## Background

Melkersson–Rosenthal syndrome (MRS) is a rare, neuro-mucocutaneous disease of unknown etiology. Age at onset varies from early childhood to late adulthood and diagnosis is based mainly on clinical detection of a triad of symptoms, such as oro-facial swelling, relapsing facial palsy and fissured tongue [1–4]. However, oligosymptomatic or monosymptomatic forms of this syndrome outnumber those with the classic triad, which is found in around one-fourth or less of patients [1–3]. Recurrent lip swelling, also termed Miescher’s syndrome or Miescher’s cheilitis granulomatosa (MCG), is the most common monosymptomatic presentation of MRS and histologic features include lymphomonocytic infiltration, non-caseating epithelioid cell granulomas, multinucleate Langerhans-type giant cells and fibrosis [2, 5].

Infectious conditions, including orofacial herpes, may precede the onset of MRS. Painless swelling, which is usually intermittent and fluctuant at the beginning, may become constant and the differential diagnosis of this sub-type of oro-facial granulomatosis includes angioedema (AE), contact dermatitis, Crohn’s disease, sarcoidosis, foreign body reaction and chronic, granulomatous infections. Diagnostic delay may be relevant, and in some cases histopathological examination of a lip biopsy is essential to diagnosis [1–3].

## Case description

**Fig. 1:**
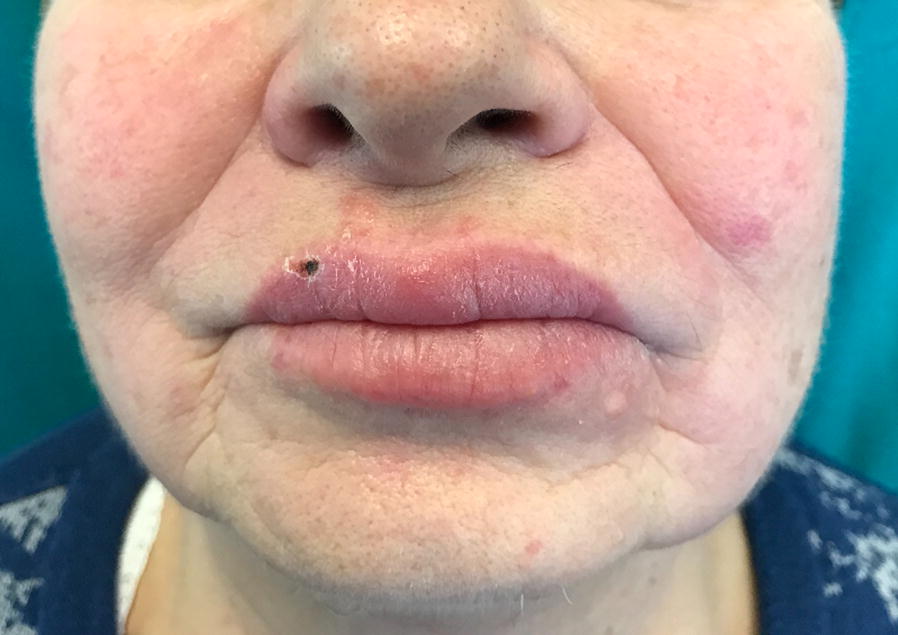
Clinical aspects. Lip swelling and healing blister upon presentation

A 71-year old, female patient with a previous history of non-Hodgkin lymphoma and transient ischemic attack came to the emergency room of our University Hospital for sudden onset of right hemifacial paraesthesia, edema of the lower lip (Fig. 1) and accentuation of an already present tinnitus. The current presentation had been preceded by a few blisters similar to those usually observed in herpes labialis, and no aphthous ulcer was detected on mouth inspection. Background therapy included aspirin and betahistine, with no personal and family history of adverse drug reactions, atopy, contact dermatitis, urticaria, angioedema, cranial nerve palsy, granulomatous or inflammatory diseases. After symptomatic treatment by intravenous steroids and antihistamines, the patient was discharged with prescription of a short-course therapy with oral prednisone and cetirizine [6]. This resulted in partial remission of symptoms, but 1 week later the patient was readmitted to ER for symptom recurrence and worsening of lip edema without detectable oral cavity and tongue alterations. Due to the apparent involvement of the 5th cranial nerve, a varicella-zoster virus (VZV) infection was hypothesized and therapy with valaciclovir initiated. On occasion, a blood sample was drawn showing evidence of anti-VZV IgG with undetectable IgM. One month later, on further admission at the ER for the same clinical picture associated with swelling over the left zygomatic region, an angioedema of unknown origin was suspected. Thus, a course of twice daily dose of 10 mg cetirizine was prescribed [6–8]. However, this approach was ineffective and also the subsequent replacement of aspirin with clopidrogel and temporary withdrawal of betahistine resulted in no improvement. IgM and eosinophil count, as well as plasma levels of angiotensin converting enzyme were in the normal range, thus helping to exclude the hypothesis of Gleich syndrome or sarcoidosis. Patch testing for dental materials was also negative, and complement screening was then performed with evidence of normal levels of circulating C1q (143 mg/L) and both antigen (302 mg/L) and functional (109%) C1-Inhibitor (C1-INH). On the contrary, C4 was low (0.03–0.04 g/L; NR 0.09–0.36 g/L) and C3 fluctuated around the lowest levels of the referral range (0.93–0.82 g/L; NR 0.9–1.8 g/L) on repeated assessments. These findings ruled out the possibility of acquired AE due C1-INH deficiency [9–11], prompting us to explore the (auto)immune-inflammatory state: antineutrophil cytoplasmic antibody tested negative, whilst low titer (1:160) anti-nuclear antibodies (ANA) were found along with antiphospholipid antibodies (lupus anticoagulants; anti-cardiolipin, anti-β^2^-glycoprotein IgM), possibly related to complement consumption [12].

**Fig. 2:**
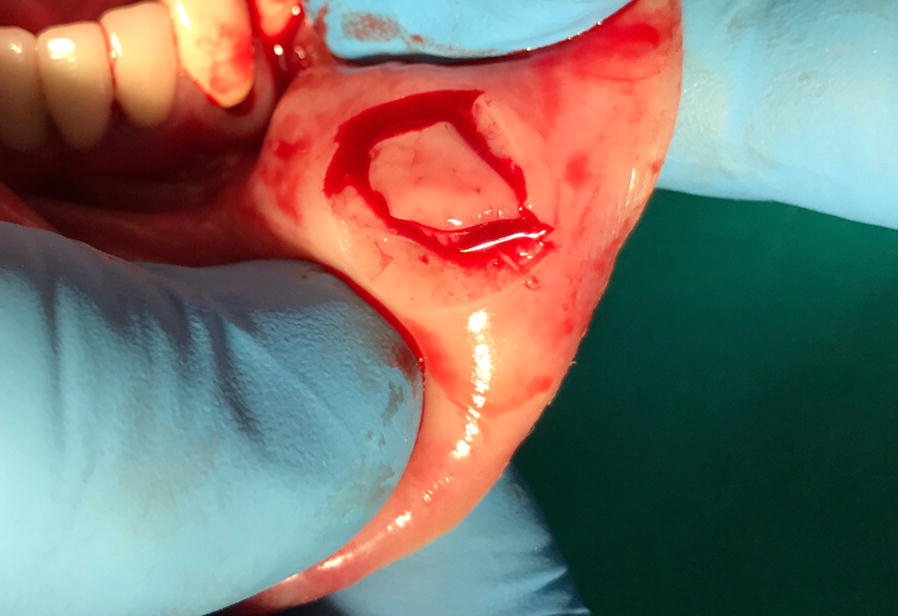
Lower lip biopsy

**Fig. 3:**
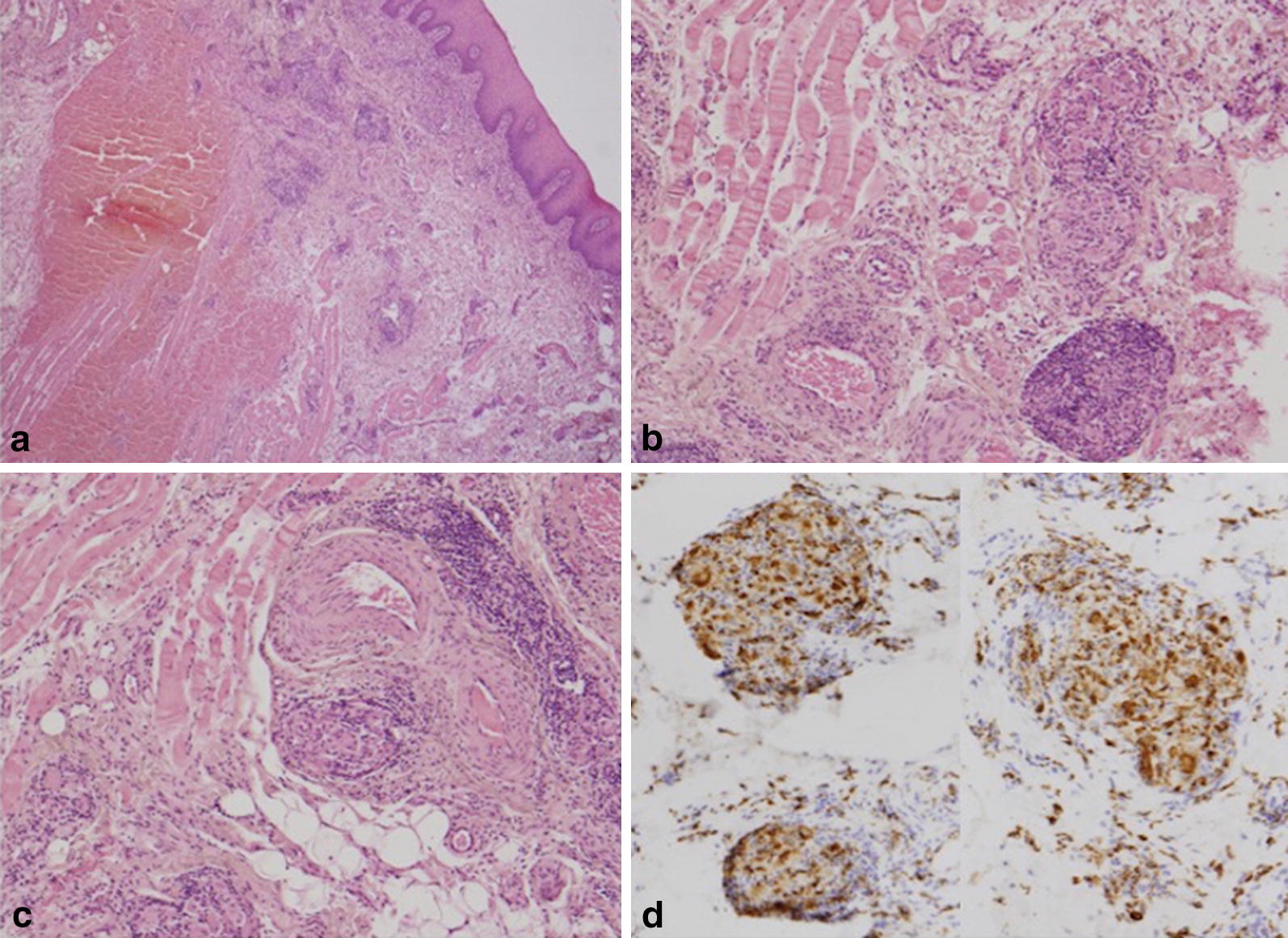
Histological aspects. **a** Lip biopsy showed the inflammatory infiltrate in the submucosa associated with numerous granulomas (H&E staining, magnification ×25). **b**, **c** Higher magnification of the granulomas in the deep submucosa, showing giant cells and lymphoplasma cells, without non-caseating necrosis (H&E staining, magnification ×80). **d** Giant cells of macrophage origin as confirmed by an antimacrophage antibody staining, CD68 (magnification ×80)

As ultrasound scan detected only a subcutaneous, hypoechogenic thickening of the inferior lip and we did not find any further clinical or laboratory sign of systemic inflammation, recurrent swelling was interpreted as a form of idiopathic angioedema and treated with tranexamic acid after a thrombophilia screen testing negative for further risk factors [10, 13]. Both this antifibrinolytic drug and a following, therapeutic course with the leukotriene receptor antagonist montelukast [7] failed to solve the edema. Finally, clinical picture, blood analyses, and lack of response to any of the previous therapies suggested the possibility of Melkersson–Rosenthal syndrome [4]. Thus, the patient was referred to the Dental Clinic, where a mucosal biopsy of the affected lower lip was performed (Fig. 2). Histopathological examination showed non caseating granulomas (Miescher’s cheilitis), consistent with a diagnosis of MRS. The two aggregates of non-caseating granulomatous inflammation consisted of lymphocytes and epithelioid histiocytes, and few multinucleated giant cells, clustered around scattered vessels (Fig. 3). Special staining for identification of fungal microorganisms and acid-fast bacteria were negative. No foreign material could be detected even at polarization. One month of oral steroid (prednisone, 25 mg qd, gradually tapered to 5 mg) resulted in remission of lip swelling but not in definitive recovery. However, since then patient’s perception of both symptoms and aesthetic relevance decreased, and at present she undertakes a few-days regimen of prednisone only when feeling a relapse of edema.

## Discussion

Melkersson–Rosenthal syndrome is a rare, neuro-mucocutaneous syndrome with an estimated incidence of 0.08% in the general population [14]. Onset of this disease is more frequent in young adults, between the second and the third decades of life [15]. The complete pattern of MRS, in which orofacial granulomatosis is accompanied by facial palsy and plicated tongue, may present in 8% to 25% of cases [16]. Some authors found oligosymptomatic forms in 50%, while the combination of orofacial swelling and facial palsy was seen in 22% by Chan et al. [2]. Elias et al., in a retrospective study found that facial edema was always present, with isolated lip involvement in 74% of cases and with only 13% of patients showing the full triad of symptoms of MRS [3]. Recurrent lip swellings presenting as a monosymptomatic, clinical variant of MRS and with identical histological features, is also known as Miescher’s cheilitis granulomatosa [2, 5]. The differential diagnosis of MRS includes a broad spectrum of heterogeneous conditions, mainly represented by other granulomatous disorders such as foreign body reaction, sarcoidosis, Crohn’s disease, Wegener’s vasculitis, amyloidosis and a wide variety of infections; Bell’s palsy, orofacial herpes, rosacea, contact dermatitis and allergic reactions should also be considered [1, 3–5]. Furthermore, when facial or lip swelling is the monosymptomatic presentation of MRS, usually intermittent upon onset as in this case report, it can strongly mimic angioedema [5, 10].

Recurrence of edema with no apparent trigger, lack of efficacy of antihistamine prophylaxis and a previous history of non-Hodgkin lymphoma prompted us to investigate the possibility of AE due to C1-INH deficiency in our patient [6–11]. Laboratory analyses did not confirm this hypothesis, nor treatment failure of both tranexamic acid and montelukast supported an alternative diagnosis of idiopathic AE [7, 8, 10, 13]. Thus, we pointed to Melkersson–Rosenthal syndrome and planned lip biopsy, which was performed on the mucosal side (Fig. 2) to avoid undesirable scarring and skin retraction.

Although MRS can be frequently diagnosed by clinical criteria with no need for further investigation [16, 17], the lack of specific biomarkers [4], the unusual age of onset in our patient, the positive anamnesis for lymphoproliferative disease, and the finding of complement consumption and autoimmune condition justified in this case an invasive approach. The histopathological examination, showing non caseating granulomas (Fig. 3), allowed to establish the correct diagnosis of MRS, probably triggered by a recent viral infection characterized by facial blistering, reassure the patient and prescribe targeted therapy. One month of oral steroid resulted in remission of lip swelling, that since then is treated by a few-days, on-demand regimen of prednisone when patient perceives a cosmetically disturbing relapse of edema.

It is well known, on the other hand, that there is no definitive therapy for MRS and recurrences are frequent [1, 5]. The proposed treatments include non-steroidal anti-inflammatory drugs, antihistamines, lymecycline and methotrexate, with systemic and/or intralesional corticosteroids usually considered as initial choices [4, 5]. Finally, a surgical approach has been suggested only for those patients with oro-facial swelling refractory to steroid therapy and/or who present a significant face deformation [1].

## Conclusion

Melkersson–Rosenthal syndrome is a rare disorder which may present as a classic triad of orofacial swelling, facial palsy and fissured tongue or, more frequently, with oligo/mono-symptomatic features. Differential diagnosis with other granulomatous diseases and angioedema must be considered, as symptoms and signs usually overlap. In selected cases, such as in this case-report of unusual presentation and with evidence of complement and immune system activation, skin or mucosal biopsy may be crucial to diagnosis.

## Abbreviations

AE: angioedema
ANA: antinuclear antibodies
C1-INH: C1-inhibitor
ER: emergency room
MCG: Miescher’s cheilitis granulomatosa
MRS: Melkersson–Rosenthal syndrome
VZV: varicella-zoster virus
